# Charge Transfer-Induced Weakening of Vibronic Coupling for Single Terrylene Molecules Adsorbed onto Hexagonal Boron Nitride

**DOI:** 10.1021/acs.jpclett.4c02899

**Published:** 2024-12-30

**Authors:** Titus de Haas, Robert Smit, Arash Tebyani, Semonti Bhattacharyya, Kenji Watanabe, Takashi Taniguchi, Francesco Buda, Michel Orrit

**Affiliations:** † Leiden Institute of Chemistry, Leiden University, 2300 RA Leiden, The Netherlands; ‡ Huygens-Kamerlingh Onnes Laboratory, Niels Bohrweg 2, 2333 CA Leiden, The Netherlands; § Research Center for Electronic and Optical Materials, National Institute for Materials Science, 1-1 Namiki, Tsukuba 305-0044, Japan

## Abstract

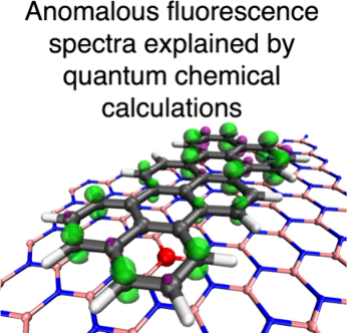

Fluorescence spectra of single terrylene molecules adsorbed on hexagonal boron nitride flakes were recorded at cryogenic temperatures. The pure electronic transitions of terrylene molecules are spread over a broad energy scale from 570 to 610 nm. Surprisingly, peaks in the vibrationally resolved fluorescence spectrum show intensity variations of ≤20-fold between molecules. We find an extreme case in which the Debye–Waller–Franck–Condon factor of the zero-phonon line exceeds 0.8. The vibronic intensity correlates with both the spectral position of the electronic transition and the frequency of the longitudinal stretch mode, which varies between 243 and 257 cm^–1^. Using density functional theory calculations, we show that these observations can be explained by terrylene chemisorption on charge-donating defect sites. The electronic states of molecules at such chemisorption sites would be very attractive for the efficient emission of single photons with narrow lines and for the generation of indistinguishable photons.

The emission of a photon by an excited molecule is generally accompanied by the release of molecular vibration quanta arising from the change in molecular geometry between the molecule’s ground and excited states. The larger the change in geometry, the more pronounced the associated vibronic bands in the fluorescence spectrum (see the theoretical discussion in section S1 of the Supporting Information). At liquid helium temperatures, these vibronic fluorescence lines become very sharp, a few inverse centimeters in width or less,^1^ and are often used as fingerprints of the single molecule under study. This so-called fluorescence line narrowing generally works well for molecules embedded in *n*-alkane Shpol’skii matrices,^2^ for specific guest molecules in organic matrices^3^ and, as recently demonstrated, for single terrylene molecules adsorbed on the surface of hexagonal boron nitride (hBN).^4^ This latter host system is also currently scrutinized for the many single-photon emitters that it can host, with emission ranging from deep ultraviolet^5^ to the near infrared.^6^ Recently, in conjunction with our work of terrylene on hBN, new insights into the potential nature of some of these emitters were gained, namely on the potential formation of aromatic molecules at the hBN/substrate interface during high-temperature annealing.^7^ The same work also summarizes the many defect structures in the hBN crystal lattice that have been proposed in the past to explain the measured emission from hBN. In addition, a recent work on hBN immersed in organic solvents showed that some solvent molecules can potentially bind to defects and display localized fluorescence, while hopping from binding site to binding site.^8^ This work is particularly interesting as it suggests that molecules can bind more strongly to specific sites on hBN.

In our previous work on single terrylene molecules on hBN, we reported that molecules with red-shifted electronic transitions displayed weaker vibronic coupling and thus show a more intense zero-phonon line in the spectrum, captured by a higher Franck–Condon–Debye–Waller factor α_FCDW_.^4^ If the interaction of the terrylene molecule with the hBN surface could be engineered to maximize this factor, this control method would potentially be much simpler than current methods such as coupling a molecule to an optical cavity.^9^ In this work, we examine potential origins of the weakened vibronic coupling, observed particularly for the most red-shifted terrylene molecules. We perform quantum chemical calculations at the density functional theory (DFT) level of the interaction of terrylene with a pristine hBN surface and find little change in the vibronic coupling. We then investigate several defects in the hBN lattice and find that some of them, in particular an oxygen substitution at a nitrogen site (O_N_) and vacancies at the boron (V_B_) or nitrogen (V_N_) site, can transfer charge to terrylene. Interestingly, we find that this charge transfer reduces the change in geometry between the ground and excited states as compared to that of terrylene in vacuum and strikingly decreases the vibronic coupling of all modes simultaneously in the emission spectrum. This charge-donating or -accepting behavior is also relevant to the field of dye-sensitized solar cells, where functionalized terrylenes and similar dyes have been employed for the efficient generation of photocurrents.^10,11^

Four examples of single-molecule fluorescence spectra obtained from four individual molecules are presented in Figure 1a–d and arranged by decreasing intensity of their vibronic lines. In all cases, we recognize, with some variation, the fingerprint of vibrational frequencies of the ground state of terrylene, which ensures that all of these molecules are terrylene (see the structure in Figure 1e) and no other impurity. Most of the lines correspond to the release of a single quantum of vibration in various intramolecular modes (0–1), with their intensity related to the Franck–Condon factor of these modes (see section S1 for a short reminder of the theory of vibronic coupling).

**Figure 1:**
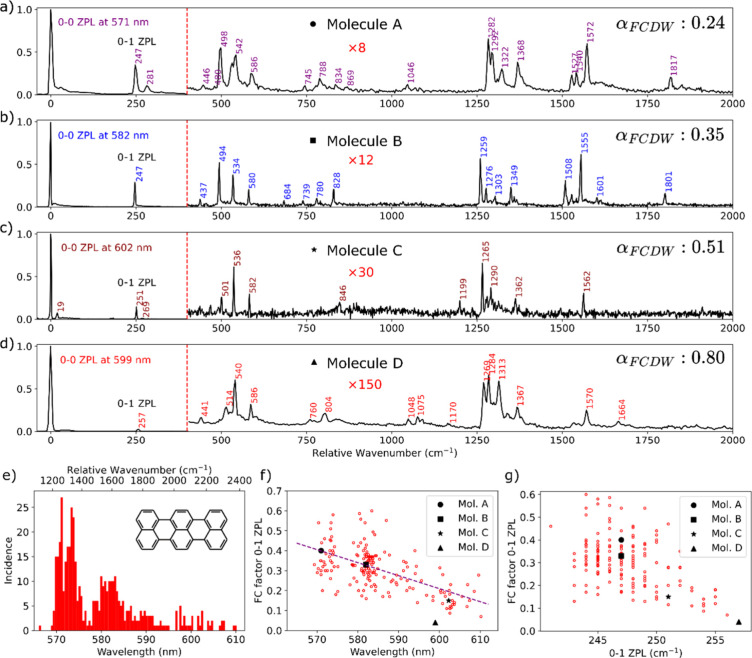
(a–d) Fluorescence emission spectra from four different molecules at different wavelengths in the inhomogeneous distribution. The spectra depicted in panels A and D were taken at a slightly lower resolution (600 lines/mm grating, 4 cm^–1^ resolution) as compared to that of the spectra depicted in panels B and C, which were taken with a grating of 1200 lines/mm (2 cm^–1^ resolution). The first three spectra show decreasing Franck–Condon factors with an increase in the 0–0 zero-phonon line (ZPL) wavelength. The fourth spectrum is the most extreme case of ultraweak vibronic coupling we could find. Note that the intensity of all vibronic lines is reduced by a factor of ∼20 with respect to molecule A. The integration times were 10 s for spectrum a, 300 s for spectrum b, 150 s for spectrum c, 600 s for spectrum d. The noise in the spectra depends on the integration time and mean signal intensities that varied for each molecule. Panel e shows the inhomogeneous distribution composed of 452 individual terrylene molecules, with the structure of terrylene in the inset. The relationship between the 0–0 ZPL and FC factor of the 0–1 ZPL is shown in panel f, while the frequency of the vibration mode responsible for the 0–1 ZPL in relation to its FC factor is shown in panel g. The corresponding molecules in the spectra of panels a–d are shown as black markers in the scatter plots. The purple dashed line in panel f is a guide to the eye for the trend, resulting from a linear fit to the data. We note the strong anticorrelation between the Franck–Condon factor and the frequency of the extensional mode at 250 cm^–1^ in panel g. This is particularly extreme for molecule D (triangle), which is the one with the highest frequency (257 cm^–1^) and the lowest 0–1 Franck–Condon factor (0.026) for this mode.

In addition to the intramolecular vibrational modes, we also see the phonon wing appearing most clearly between 0 and 100 cm^–1^ to the red of the 0–0 ZPL origin. The intensity of the vibronic fingerprint decreases when going from molecule A to D, with the corresponding values of the Franck–Condon–Debye–Waller factor (α_FCDW_) being 0.24 (A), 0.35 (B), 0.51 (C), and 0.80 (D). Molecule D shows an extremely weak vibronic coupling, with a Franck–Condon–Debye–Waller factor of at least 0.80, which largely exceeds the largest ones observed for organic molecules in a matrix at low temperatures. To the best of our knowledge, all factors observed so far for organic molecules were <55%.^12^ Another surprising observation is that, for each molecule, the coupling strengths of all vibrational modes, including lattice phonons, vary in a similar way across the spectrum. An alternative formulation of this fact is that the change in the geometry of some particular molecules upon excitation can become very small for all molecular modes simultaneously. This is surprising because, whereas we could expect a weak equilibrium shift for certain modes due to specific interaction with the substrate, we would not expect this to occur simultaneously for all of the modes.

Figure 1e presents a histogram of the 0–0 ZPL spectral positions of 452 molecules. The broad distribution in this histogram, extending over a particularly broad range (570–610 nm or >1000 wavenumbers), exceeds what is usually observed for terrylene embedded in molecular crystals and presents distinct maxima ∼10 cm^–1^ in width. We assign this structure in the distribution of Figure 1e, with maxima around 571, 582, and 600 nm, to selection within a broad profile through resonance of vibronic lines with the excitation laser. As all molecules were excited with a fixed 532 nm laser, molecules with absorption lines at this wavelength had an increased probability of being selected in the histogram. Indeed, the relative position in wavenumber of the 0–0 zero-phonon lines (ZPLs) with respect to the laser wavelength resembles the mirror image of the vibrational spectra of Figure 1a–d. The large inhomogeneous broadening may be the result of significant variations in the environment around the molecule, which might be affected by parameters such as significant tensile strain^13^ or the many types of (charged) defects present.^14^ In addition, the annealing that we perform prior to the deposition of molecules on the hBN may also hydroxylate parts of the hBN^15^ or create wrinkles in the sheets.^16^

We now turn to the scatter plots of panels f and g of Figure 1. We observe a clear correlation in Figure 1f between the spectral position of a molecule and the intensity of its first vibronic line at ≈250 cm^–1^, corresponding to stretching of terrylene along its long axis. Red-shifted molecules tend to be less strongly coupled to vibrations. The correlation is even more pronounced in Figure 1g when the vibronic coupling of the longitudinal stretch mode is correlated with its frequency. Molecules with a high stretch frequency appear clearly more weakly coupled to the electronic system. This correlation reaches an extreme point for molecule D, whose FC factor for the 0–1 ZPL (0.027) is >10 times lower than the average over all other molecules, while its stretch mode is that with the highest frequency at 257 cm^–1^. As the effective mass of this skeleton mode is not likely to change much from molecule to molecule, the high frequency points to a fairly strong interaction of molecule D with the surface, which appears to change the intramolecular spring constant by ∼8%.

As noted above for molecule D, we see that the level of coupling to all vibrations decreases in a similar way when going from molecule A to D. Although some minor intensity changes, shifts, and splitting of certain modes can be spotted, we observe no major redistribution of intensities between the main modes, so that all of them follow qualitatively the general trend observed on the 250 cm^–1^ stretch mode.

DFT was employed to study physisorption of terrylene on a pristine hBN surface and on hBN, including a selection of potential lattice defects. The considered defects include a boron vacancy (V_B_), a nitrogen vacancy (V_N_), the boron nitride (V_BN_) divacancy, and oxygen substitution at the boron (O_B_) or nitrogen (O_N_) centers. Indeed, as our samples were annealed in a moderate vacuum of residual air, oxygen substitution appears to be plausible. Although a comprehensive study of possible defects is beyond the scope of this work, these vacancies and substitutions are representative of a wider range of defects with either electron-accepting or electron-donating properties. Ground state structure optimizations and band structure calculations of the terrylene–hBN interfaces were performed at the HSE06+D3BJ DFT level using the CP2K software package.^17−19^ Calculations of the vibronic spectra were subsequently performed with FCclasses3 using geometries, Hessians, and dipole moments calculated with Gaussian.^20,21^ In these excited state calculations, a two-layer ONIOM(B3LYP/PM3) approach was employed, as considering the entire interface structure at the time-dependent DFT (TD-DFT) level was too computationally demanding.^22−30^ More details about the computational workflow are provided in sections S2 and S3 of the Supporting Informarion.

The first two columns of Table 1 present physisorption energies and partial Mulliken charge accumulation on terrylene in the presence of the considered defects, calculated at the full DFT level. Charge analysis has also been performed using Hirshfeld and intrinsic orbital analysis charges, which show the same trend and are listed in Table S2.^31^ The physisorption energy of terrylene on a perfect monolayer of hBN was found to be −2.44 eV, compared to that of terrylene and hBN in a vacuum. This adsorption energy follows the trend reported in the literature of increasing adsorption energy with an increase in molecule size for polycyclic aromatic hydrocarbons (PAHs) on hBN.^32^ Defect site binding energies were established by comparing the adsorption energies on defects with the adsorption energy on the defect-free lattice. It was found that the V_BN_ divacancy and the O_B_ defects bind the molecule less strongly than pristine hBN, with values of 0.08 and 0.15 eV, respectively. It is therefore unlikely that the terrylene molecule would be localized on these specific defects. On the contrary, the adsorption energies in the presence of the V_N_ and V_B_ vacancies are slightly higher (by 0.02 and 0.08 eV, respectively) than those of the defect-free case, and specifically for the O_N_ defect, the binding is much stronger (by 1.52 eV). Notably, this latter defect has also been proposed as a source for specific features observed via X-ray spectroscopy.^33^ The reported partial Mulliken charges reveal that the three terrylene-adsorbing defects (V_N_, V_B_, and O_N_) donate a considerable amount of electron (V_N_ and O_N_) or hole (V_B_) density to terrylene. This type of charge transfer has been reported before in GGA-based DFT studies on hBN–graphene heterostructures.^34^ As a consequence of this charge transfer, we observe significant changes in the extent to which terrylene elongates or contracts upon excitation from the ground state to the excited state. For instance, in the defect-free hBN, the long-axis contraction in the excited state is −0.035 Å, whereas in the presence of the O_N_ defect, this contraction is reduced to only −0.012 Å (see Table 1). Therefore, one can expect the intensity of vibrational modes coupled to the electronic transition to be affected considerably in the fluorescence spectrum.

**Table 1:** Adsorption Energies and Properties of Terrylene Adsorbed on Different Defects^a^

			long-axis length (Å)			short-axis length (Å)		
	adsorption energy (eV)	Mulliken charge on terrylene (atomic units)	ground state	excited state	Δ*x*	ground state	excited state	Δ*x*
vacuum	–	–	10.078	10.044	–0.034	4.839	4.883	0.044
pristine hBN	–2.44	0.02	10.089	10.054	–0.035	4.843	4.887	0.044
V_BN_^b^	–2.36	0.02	–	–	–	–	–	–
V_B_	–2.52	0.83	10.054	10.041	–0.013	4.829	4.854	0.023
V_N_	–2.46	–0.68	10.094	10.082	–0.012	4.839	4.860	0.021
O_B_^b^	–2.29	0.01	–	–	–	–	–	–
O_N_	–3.96	–0.70	10.095	10.083	–0.012	4.839	4.860	0.021

a Note that adsorption energies and Mulliken charges are calculated at the full DFT level, while the structural properties are calculated at the ONIOM(B3LYP/PM3) level. Δ*x* indicates the change in axis length from the excited to ground state.

b As the V_BN_ and O_B_ defects have a lower binding energy for the terrylene molecule compared to defect-free hBN, we did not further investigate the excited state geometry of terrylene on these defects.

Figure 2a shows the optimized geometry of terrylene on the hBN single-layer lattice. The two structures are nearly commensurate. The terrylene carbon atoms of the graphene A sublattice are found to reside on top of monolayer boron atoms, whereas the terrylene carbon atoms of the B sublattice are placed over the centers of the (BN)_3_ hexagons (A and B sublattices are indicated in Figure 2a). The hBN nitrogens are thus placed underneath the centers of the terrylene aromatic rings. Previous theoretical works have also found this configuration to be most stable for similar PAHs on hBN.^32,35^ Our TD-DFT calculations show that the experimentally observed fluorescence at ∼600 nm is associated with the electronic transition that involves predominantly (>70%) the HOMO and LUMO orbitals. Panels b and c of Figure 2 present the HOMO and LUMO orbitals of the terrylene molecule on the hBN surface. Both orbitals are localized on the terrylene molecule and do not hybridize with hBN electronic states. This is in line with Figure 2f, which shows that the frontier orbitals of terrylene are found to reside completely isolated within the hBN bandgap. The calculated bandgap of ∼5.7 eV is similar to previous studies reporting hBN bandgaps of ∼6 eV.^35,36^ The shapes of the HOMO and LUMO orbitals provide a physical interpretation for the observed short-axis elongation and long-axis contraction when terrylene is excited from the ground state to the excited state as electron density is transferred from bonding orbitals along the short axis to bonding orbitals along the long axis. Similarly, one can expect that populating the LUMO orbital may affect the spectral position of the long-axis vibration observed at ≈250 cm^–1^.

**Figure 2:**
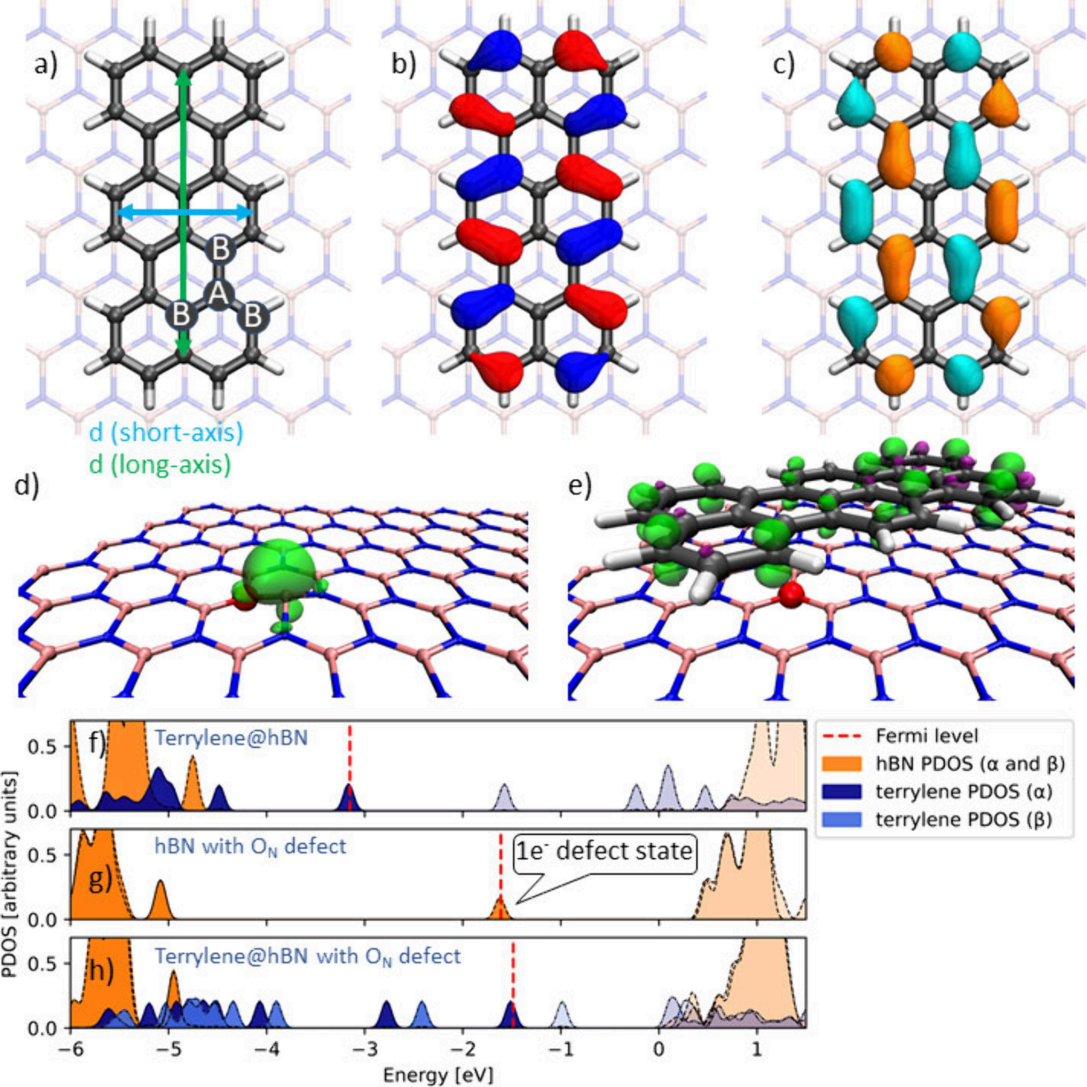
(a–c) Terrylene molecules adsorbed on a single monolayer of pristine hBN. (a) Longitudinal (green) and short (blue) axes and (b) HOMO and (c) LUMO, with the isosurfaces plotted at 0.03 au. The carbon atoms of the graphene A and B sublattices of terrylene are depicted with insets in panel a. (d and e) Spin density on the O_N_ chemisorption site before and after binding of terrylene with an isosurface plotted at 0.005 au. Projected density of states (PDOS) of (f) terrylene adsorbed on pristine hBN, (g) hBN with the O_N_ defect, and (h) terrylene adsorbed on hBN with the O_N_ defect. The PDOS values were computed in CP2K at the HSE06+D3(BJ) level and include a Gaussian broadening of 0.05 eV. Bands with transparent colors correspond to unoccupied levels. The α and β bands for hBN with the O_N_ defect in panels g and h largely overlap and are depicted in the same color (orange). However, it should be noted that the defect state in panel g is singly occupied.

For the remainder of this discussion, we focus on the terrylene interaction with the O_N_ defect as binding to this defect was found to be particularly strong. Figure 2d shows the spin density at the O_N_ defect site. Interestingly, the excess electron is localized almost entirely on the lone pair of the boron atom adjacent to the introduced oxygen atom, resulting in an out-of-plane distortion of the boron atom. Figure 2g shows that the introduced defect state is localized within the hBN bandgap, at an energy similar to that of the terrylene LUMO orbital. Upon chemisorption of terrylene, the excess electron is transferred to the terrylene, which is made apparent by the spin density in Figure 2e and the PDOS plot in Figure 2h. Once the excess electron has transferred to terrylene, the hBN relaxes again to its planar structure. A similar electron donor state is introduced by the V_N_ vacancy, which is shown in Figure S2. We note that the terrylene HOMO–LUMO gap decreases upon charge transfer. This aligns with the red-shift of the 0–0 ZPL that was observed for specific terrylene molecules in the single-molecule fluorescence experiments. In contrast to the O_N_ and V_N_ cases, the V_B_ vacancy introduces an acceptor state close to the valence band. In this case, it is the terrylene molecule that donates charge to the hBN surface rather than the opposite (see Figure S3). Despite considerable charge transfer at all of these chemisorption sites, no single isolated covalent bond is formed between the adsorbate and adsorber. Only an ∼0.2 Å shortening of the terrylene–hBN distance is observed in the case of chemisorption on the O_N_ and V_N_ defects (see Table S3).

In addition to the geometry optimizations, PBE-DFT-based molecular dynamics simulations were carried out to gain insights into the diffusive behavior of terrylene on pristine hBN. The optimized structure was used as input for a 5 ps equilibration run at 300 K. Subsequently, the system was propagated for an additional 5 ps in a production run. Figure S4 shows three structures sampled from the MD production run trajectory at 0, 2, and 4 ps. Despite the relatively high binding energy, the MD simulations show that at 300 K, terrylene can rotate and even translate on the pristine hBN monolayer on the picosecond time scale. This strengthens our belief that the observed localized emission at room temperature^37^ originates from terrylene chemisorbed at specific defect sites.

Figure 3 shows the calculated vibrationally resolved fluorescence spectra for terrylene in vacuum, terrylene on hBN, and terrylene adsorbed onto the O_N_ defect site. Both the spectra of terrylene in a vacuum and terrylene on pristine hBN match very closely the experimental spectrum reported for molecule A in Figure 1a. The most prominent feature in all simulated spectra is the peak observed at ∼250 cm^–1^, which originates from a stretching vibration along the terrylene longitudinal axis (Figure S6, mode 11).^39,40^ Other strong peaks in the spectrum are found around ∼1285 and ∼1570 cm^–1^ and also involve nuclear distortions in the same direction and with the same A_g_ symmetry (Figure S6, modes 86 and 108). The peaks around 540 cm^–1^ are associated with a contraction along the terrylene short axis and preserve the same A_g_ symmetry. Table S4 provides a complete assignment of the fluorescence signals with an intensity of >1% of the 0–0 ZPL intensity. Our interpretation aligns with that of Deperasińska and Kozankiewicz^39^ and that of Greiner and Sundholm.^40^

**Figure 3:**
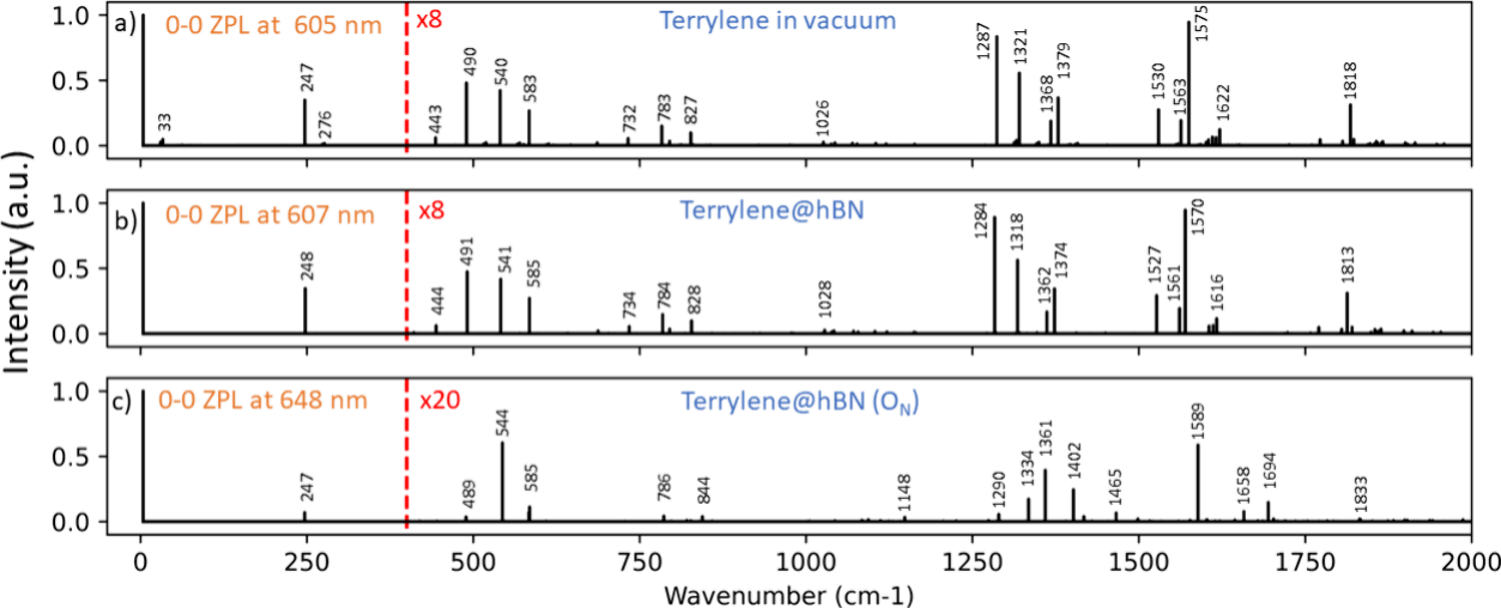
Computed vibrationally resolved fluorescence spectra of terrylene (a) in vacuum and on (b) hBN and (c) hBN adsorbed onto the O_N_ defect. All spectra are shifted such that their respective 0–0 ZPL is centered at 0. The spectra are corrected with a linear scaling function with parameters of 0.977 and 4.132, as suggested by Palafox.^38^ All intensities are scaled such that the intensity of the 0–0 ZPL equals 1. The intensities of the peaks observed above 400 cm^–1^ are multiplied by a factor of 8 for panels a and b and a factor of 20 for panel c.

None of the vibrational peak intensities change significantly between the terrylene spectrum in vacuum and the terrylene spectrum on pristine hBN. We thus conclude that the observed ultraweak vibronic coupling cannot be attributed solely to the adsorption process of terrylene on the defect-free lattice. However, both the fluorescence wavelength and the intensity of features in the spectrum change drastically when terrylene is chemisorbed on the O_N_ defect site or on the V_N_ and V_B_ sites (see Figure S5). The effective charge transfer and consequential structural rearrangement in the ground and excited state led to a much weaker vibronic coupling, characterized by an overall diminished intensity of all vibrational features and an increased intensity of the 0–0 ZPL. The intensity ratio of the stretching mode around 250 cm^–1^ to the ZPL decreases by a factor of ≈5 when comparing the terrylene on defect-free hBN with the terrylene on the O_N_ defect (see Tables S5 and S6). Interestingly, peaks that originate from vibrational overtones and combinations of vibrational modes are weakened substantially more than peaks that originate from a single mode in its lowest vibrational state, as demonstrated by the peaks at ∼490 and ∼1817 cm^–1^ that almost vanish in spectrum c of Figure 3. This diminishing of overtone peaks is also observed in the experimental spectra in panels c and d of Figure 1. The observation that all peaks are simultaneously diminished is explained by the fact that all visible vibrational modes have the same symmetry and involve nuclear displacements along the terrylene short and long axes (Figure S6). The wavelength of the 0–0 ZPL shifts to the red by ∼43 nm upon introduction of the O_N_ and V_N_ defects (see Figure 3 and Figure S5), closely matching the experimentally observed red-shift. The adsorption of terrylene on the hole-donating V_B_ vacancy also leads to a diminished vibronic coupling, but to a much larger, ∼100 nm, red-shift of the 0–0 ZPL (see Figure S5). Even though this red-shift is larger than the experimentally observed red-shift, we do not rule out the possibility of electron-withdrawing defects affecting the fluorescence spectrum, as the overly large shift could be an artifact of exaggerated charge transfer description within the ONIOM approach. In summary, the significant overall reduction of vibronic coupling, the nearly vanishing vibrational overtones, and the red-shifted 0–0 ZPL observed for terrylene on charge-donating defects strongly suggest that such a defect may be responsible for the experimentally observed large molecule-to-molecule variations. In the work of Vasilev et al.,^41^ large shifts of a 0–0 ZPL were also observed upon localized charging of a phthalocyanine molecule through deprotonation. These shifts were interpreted as an “internal Stark effect”. In our case, the interaction of terrylene with a particular hBN defect also leads to charge transfer from the defect to the aromatic molecule and therefore to local electric fields shifting the electronic transition. These effects are naturally included in the DFT calculations that reproduce the red-shift of the 0–0 ZPL. In a similar manner, the change in vibronic coupling intensity, which we observe experimentally and confirm through DFT calculations, could be interpreted as a vibrational Stark effect, i.e., a change in coupling due to charge transfer-induced local fields.

In summary, the single terrylene molecules observed through fluorescence on an hBN surface do not diffuse translationally or rotationally at low temperatures and even show localized emission at room temperature, as also noted in earlier experiments.^4,37^ The latter observation is in contrast to the performed molecular dynamics simulations that show that molecules would diffuse rapidly on defect-free hBN. Moreover, many molecules display a comparatively strong red-shift of their optical transition (from 570 to >620 nm) and considerable variation in the intensity of their vibronic structure in fluorescence. All of these observations point to a strong interaction of the molecule with the substrate, although the interaction is not strong enough to radically modify the chemical nature of terrylene. As van der Waals interactions of terrylene with hBN are not strong enough to account for the observations described above, it is natural to assume that some degree of chemisorption between the molecule and the surface takes place. From quantum chemical calculations, we can select two potential candidate binding sites that are given by chemisorption to a O_N_ or V_N_ defect assisted by charge transfer from the defect to terrylene. Moreover, the binding to such defects affects the geometries of terrylene in the ground and excited states in such a way that the vibronic couplings of all modes are affected simultaneously, as observed in the experiment. To experimentally investigate the possibility of specific defects being responsible for chemisorption of terrylene, we propose experiments that involve the engineering of defects, such as oxygen-related defects created by hydrogen plasma irradiation^42^ or vacancies by low-energy ion irradiation.^43^

## Supporting Information Available

Brief summary of vibronic coupling theory, experimental and computational details, and additional results of quantum chemical calculations (PDF)
Transparent Peer Review report available (PDF)

## References

[ref1] Zirkelbach J.; Mirzaei M.; Deperasińska I.; Kozankiewicz B.; Gurlek B.; Shkarin A.; Utikal T.; Götzinger S.; Sandoghdar V. High-Resolution Vibronic Spectroscopy of a Single Molecule Embedded in a Crystal. J. Chem. Phys. 2022, 156 (10), 104301 10.1063/5.0081297. 35291792

[ref2] Gooijer C.; Ariese F.; Hofstraat J. W. Shpol’skii Spectroscopy and Other Site-Selection Methods. Applications in Environmental Analysis, Bioanalytical Chemistry, and Chemical Physics; Chemical Analysis; Wiley and Sons: New York, 2000.

[ref3] Adhikari S.; Smit R.; Orrit M. Future Paths in Cryogenic Single-Molecule Fluorescence Spectroscopy. J. Phys. Chem. C 2024, 128 (1), 3–18. 10.1021/acs.jpcc.3c06564. PMC10788914 38229590

[ref4] Smit R.; Tebyani A.; Hameury J.; van der Molen S. J.; Orrit M. Sharp Zero-Phonon Lines of Single Organic Molecules on a Hexagonal Boron-Nitride Surface. Nat. Commun. 2023, 14 (1), 7960 10.1038/s41467-023-42865-4. 38042826 PMC10693553

[ref5] Bourrellier R.; Meuret S.; Tararan A.; Stéphan O.; Kociak M.; Tizei L. H. G.; Zobelli A. Bright UV Single Photon Emission at Point Defects in H-BN. Nano Lett. 2016, 16 (7), 4317–4321. 10.1021/acs.nanolett.6b01368. 27299915

[ref6] Camphausen R.; Marini L.; Tawfik S. A.; Tran T. T.; Ford M. J.; Palomba S. Observation of Near-Infrared Sub-Poissonian Photon Emission in Hexagonal Boron Nitride at Room Temperature. APL Photonics 2020, 5 (7), 076103 10.1063/5.0008242.

[ref7] Neumann M.; Wei X.; Morales-Inostroza L.; Song S.; Lee S.-G.; Watanabe K.; Taniguchi T.; Götzinger S.; Lee Y. H. Organic Molecules as Origin of Visible-Range Single Photon Emission from Hexagonal Boron Nitride and Mica. ACS Nano 2023, 17 (12), 11679–11691. 10.1021/acsnano.3c02348. 37276077

[ref8] Ronceray N.; You Y.; Glushkov E.; Lihter M.; Rehl B.; Chen T.-H.; Nam G.-H.; Borza F.; Watanabe K.; Taniguchi T.; Roke S.; Keerthi A.; Comtet J.; Radha B.; Radenovic A. Liquid-Activated Quantum Emission from Pristine Hexagonal Boron Nitride for Nanofluidic Sensing. Nat. Mater. 2023, 22 (10), 1236–1242. 10.1038/s41563-023-01658-2. 37652991 PMC10533396

[ref9] Wang D.; Kelkar H.; Martin-Cano D.; Rattenbacher D.; Shkarin A.; Utikal T.; Götzinger S.; Sandoghdar V. Turning a Molecule into a Coherent Two-Level Quantum System. Nat. Phys. 2019, 15 (5), 483–489. 10.1038/s41567-019-0436-5.

[ref10] Edvinsson T.; Pschirer N.; Schöneboom J.; Eickemeyer F.; Boschloo G.; Hagfeldt A. Photoinduced Electron Transfer from a Terrylene Dye to TiO2: Quantification of Band Edge Shift Effects. Chem. Phys. 2009, 357 (1), 124–131. 10.1016/j.chemphys.2008.11.024.

[ref11] Monti A.; Negre C. F. A.; Batista V. S.; Rego L. G. C.; de Groot H. J. M.; Buda F. Crucial Role of Nuclear Dynamics for Electron Injection in a Dye–Semiconductor Complex. J. Phys. Chem. Lett. 2015, 6 (12), 2393–2398. 10.1021/acs.jpclett.5b00876. 26266622

[ref12] Schofield R. C.; Burdekin P.; Fasoulakis A.; Devanz L.; Bogusz D. P.; Hoggarth R. A.; Major K. D.; Clark A. S. Narrow and Stable Single Photon Emission from Dibenzoterrylene in Para-Terphenyl Nanocrystals. ChemPhysChem 2022, 23 (4), e202100809 10.1002/cphc.202100809. 34905640 PMC9302619

[ref13] Mendelson N.; Doherty M.; Toth M.; Aharonovich I.; Tran T. T. Strain-Induced Modification of the Optical Characteristics of Quantum Emitters in Hexagonal Boron Nitride. Adv. Mater. 2020, 32 (21), 1908316 10.1002/adma.201908316. 32270896

[ref14] Wong D.; Velasco J.; Ju L.; Lee J.; Kahn S.; Tsai H.-Z.; Germany C.; Taniguchi T.; Watanabe K.; Zettl A.; Wang F.; Crommie M. F. Characterization and Manipulation of Individual Defects in Insulating Hexagonal Boron Nitride Using Scanning Tunnelling Microscopy. Nat. Nanotechnol. 2015, 10 (11), 949–953. 10.1038/nnano.2015.188. 26301901

[ref15] Ren J.; Stagi L.; Innocenzi P. Hydroxylated Boron Nitride Materials: From Structures to Functional Applications. J. Mater. Sci. 2021, 56 (6), 4053–4079. 10.1007/s10853-020-05513-6.

[ref16] Zhang G.; Chang Y.; Yan B. The Study of the Wrinkles of Hexagonal Boron-Nitride Flake after the Annealing. Crystals 2023, 13 (2), 304 10.3390/cryst13020304.

[ref17] Heyd J.; Scuseria G. E.; Ernzerhof M. Hybrid Functionals Based on a Screened Coulomb Potential. J. Chem. Phys. 2003, 118 (18), 8207–8215. 10.1063/1.1564060.

[ref18] Heyd J.; Scuseria G. E. Efficient Hybrid Density Functional Calculations in Solids: Assessment of the Heyd–Scuseria–Ernzerhof Screened Coulomb Hybrid Functional. J. Chem. Phys. 2004, 121 (3), 1187–1192. 10.1063/1.1760074. 15260659

[ref19] Kühne T. D.; Iannuzzi M.; Del Ben M.; Rybkin V. V.; Seewald P.; Stein F.; Laino T.; Khaliullin R. Z.; Schütt O.; Schiffmann F.; Golze D.; Wilhelm J.; Chulkov S.; Bani-Hashemian M. H.; Weber V.; Borštnik U.; Taillefumier M.; Jakobovits A. S.; Lazzaro A.; Pabst H.; Müller T.; Schade R.; Guidon M.; Andermatt S.; Holmberg N.; Schenter G. K.; Hehn A.; Bussy A.; Belleflamme F.; Tabacchi G.; Glöß A.; Lass M.; Bethune I.; Mundy C. J.; Plessl C.; Watkins M.; VandeVondele J.; Krack M.; Hutter J. CP2K: An Electronic Structure and Molecular Dynamics Software Package - Quickstep: Efficient and Accurate Electronic Structure Calculations. J. Chem. Phys. 2020, 152 (19), 194103 10.1063/5.0007045. 33687235

[ref20] Cerezo J.; Santoro F. *FCclasses3* : Vibrationally-resolved Spectra Simulated at the Edge of the Harmonic Approximation. J. Comput. Chem. 2023, 44 (4), 626–643. 10.1002/jcc.27027. 36380723 PMC10100349

[ref21] Frisch M. J.; Trucks G. W.; Schlegel H. B.; Scuseria G. E.; Robb M. A.; Cheeseman J. R.; Scalmani G.; Barone V.; Petersson G. A.; Nakatsuji H.; Li X.; Caricato M.; Marenich A. V.; Bloino J.; Janesko B. G.; Gomperts R.; Mennucci B.; Hr D. J. Gaussian 16; Gaussian, Inc.: Wallingford, CT, 2016.

[ref22] Dapprich S.; Komaromi I.; Byun K. S.; Morokuma K.; Frisch M. J. A New ONIOM Implementation in Gaussian98. Part I. The Calculation of Energies, Gradients, Vibrational Frequencies and Electric FIeld Derivatives; 1999.

[ref23] Chung L. W.; Sameera W. M. C.; Ramozzi R.; Page A. J.; Hatanaka M.; Petrova G. P.; Harris T. V.; Li X.; Ke Z.; Liu F.; Li H.-B.; Ding L.; Morokuma K. The ONIOM Method and Its Applications. Chem. Rev. 2015, 115 (12), 5678–5796. 10.1021/cr5004419. 25853797

[ref24] Becke A. D. A New Mixing of Hartree-Fock and Local Density-Functional Theories. J. Chem. Phys. 1993, 98 (2), 1372–1377. 10.1063/1.464304.

[ref25] Vosko S. H.; Wilk L.; Nusair M. Accurate Spin-Dependent Electron Liquid Correlation Energies for Local Spin Density Calculations: A Critical Analysis. Can. J. Phys. 1980, 58 (8), 1200–1211. 10.1139/p80-159.

[ref26] Lee C.; Yang W.; Parr R. G. Development of the Colle-Salvetti Correlation-Energy Formula into a Functional of the Electron Density. Phys. Rev. B 1988, 37 (2), 785–789. 10.1103/PhysRevB.37.785. 9944570

[ref27] Stephens P. J.; Devlin F. J.; Chabalowski C. F.; Frisch M. J. Ab Initio Calculation of Vibrational Absorption. J. Phys. Chem. 1994, 98 (45), 11623–11627. 10.1021/j100096a001.

[ref28] Stewart J. J. P. Optimization of Parameters for Semiempirical Methods I. Method. J. Comput. Chem. 1989, 10 (2), 209–220. 10.1002/jcc.540100208.

[ref29] Stewart J. J. P. Optimization of Parameters for Semiempirical Methods II. Applications. J. Comput. Chem. 1989, 10 (2), 221–264. 10.1002/jcc.540100209.

[ref30] Stewart J. J. P. Optimization of Parameters for Semiempirical Methods. III Extension of PM3 to Be, Mg, Zn, Ga, Ge, As, Se, Cd, In, Sn, Sb, Te, Hg, Tl, Pb, and Bi. J. Comput. Chem. 1991, 12 (3), 320–341. 10.1002/jcc.540120306.

[ref31] Knizia G. Intrinsic Atomic Orbitals: An Unbiased Bridge between Quantum Theory and Chemical Concepts. J. Chem. Theory Comput. 2013, 9 (11), 4834–4843. 10.1021/ct400687b. 26583402

[ref32] Chen X.; Jia S.; Ding N.; Shi J.; Wang Z. Capture of Aromatic Organic Pollutants by Hexagonal Boron Nitride Nanosheets: Density Functional Theoretical and Molecular Dynamic Investigation. Environ. Sci.: Nano 2016, 3 (6), 1493–1503. 10.1039/C6EN00378H.

[ref33] McDougall N. L.; Partridge J. G.; Nicholls R. J.; Russo S. P.; McCulloch D. G. Influence of Point Defects on the near Edge Structure of Hexagonal Boron Nitride. Phys. Rev. B 2017, 96 (14), 144106 10.1103/PhysRevB.96.144106.

[ref34] Prasad M. K.; Al-Ani O. A.; Goss J. P.; Mar J. D. Charge Transfer Due to Defects in Hexagonal Boron Nitride/Graphene Heterostructures: An *Ab Initio* Study. Phys. Rev. Materials 2023, 7 (9), 094003 10.1103/PhysRevMaterials.7.094003.

[ref35] Melani G.; Guerrero-Felipe J. P.; Valencia A. M.; Krumland J.; Cocchi C.; Iannuzzi M. Donors, Acceptors, and a Bit of Aromatics: Electronic Interactions of Molecular Adsorbates on hBN and MoS _2_ Monolayers. Phys. Chem. Chem. Phys. 2022, 24 (27), 16671–16679. 10.1039/D2CP01502A. 35766517

[ref36] Elias C.; Valvin P.; Pelini T.; Summerfield A.; Mellor C. J.; Cheng T. S.; Eaves L.; Foxon C. T.; Beton P. H.; Novikov S. V.; Gil B.; Cassabois G. Direct Band-Gap Crossover in Epitaxial Monolayer Boron Nitride. Nat. Commun. 2019, 10 (1), 2639 10.1038/s41467-019-10610-5. 31201328 PMC6572751

[ref37] Han S.; Qin C.; Song Y.; Dong S.; Lei Y.; Wang S.; Su X.; Wei A.; Li X.; Zhang G.; Chen R.; Hu J.; Xiao L.; Jia S. Photostable Fluorescent Molecules on Layered Hexagonal Boron Nitride: Ideal Single-Photon Sources at Room Temperature. J. Chem. Phys. 2021, 155 (24), 244301 10.1063/5.0074706. 34972379

[ref38] Palafox M. A. DFT Computations on Vibrational Spectra: Scaling Procedures to Improve the Wavenumbers. Physical Sciences Reviews 2018, 3 (6), 20170184 10.1515/psr-2017-0184.

[ref39] Deperasińska I.; Kozankiewicz B. Non-Planar Distortion of Terrylene Molecules in a Naphthalene Crystal. Chem. Phys. Lett. 2017, 684, 208–211. 10.1016/j.cplett.2017.06.043.

[ref40] Greiner J.; Sundholm D. Calculation of Vibrationally Resolved Absorption and Fluorescence Spectra of the Rylenes. Phys. Chem. Chem. Phys. 2020, 22 (4), 2379–2385. 10.1039/C9CP06089H. 31935005

[ref41] Vasilev K.; Doppagne B.; Neuman T.; Rosławska A.; Bulou H.; Boeglin A.; Scheurer F.; Schull G. Internal Stark Effect of Single-Molecule Fluorescence. Nat. Commun. 2022, 13 (1), 677 10.1038/s41467-022-28241-8. 35115513 PMC8813982

[ref42] Xiao Y.; Yu H.; Wang H.; Zhu X.; Chen L.; Gao W.; Liu C.; Yin H. Defect Engineering of Hexagonal Boron Nitride Nanosheets via Hydrogen Plasma Irradiation. Appl. Surf. Sci. 2022, 593, 153386 10.1016/j.apsusc.2022.153386.

[ref43] Längle M.; Mayer B. M.; Madsen J.; Propst D.; Bo A.; Kofler C.; Hana V.; Mangler C.; Susi T.; Kotakoski J. Defect-Engineering Hexagonal Boron Nitride Using Low-Energy Ar+ Irradiation. arXiv 2024, 10.48550/arXiv.2404.07166.

