# Supplementary material for: Charge Transfer-Induced Weakening of Vibronic Coupling for Single Terrylene Molecules Adsorbed onto Hexagonal Boron Nitride
Source: J Phys Chem Lett. 2024 Dec 30;16(1):349–56. doi: 10.1021/acs.jpclett.4c02899 (PMC11726798; doi:10.1021/acs.jpclett.4c02899)
Supplement: Supplementary file 1 — jz4c02899_si_001.pdf [file jz4c02899_si_001.pdf]

SUPPORTING INFORMATION for

Charge Transfer-Induced Weakening of Vibronic Coupling for Single Terrylene Molecules Adsorbed onto hBN

Titus de Haas[1†], Robert Smit[2†], Arash Tebyani[2], Semonti Bhattacharyya[2], Kenji Watanabe[3], Takashi Taniguchi[3], Francesco Buda[1*], Michel Orrit[2*]

[1] Leiden Institute of Chemistry, Leiden University, 2300 RA Leiden, The Netherlands

[2] Huygens-Kamerlingh Onnes Laboratory, Niels Bohrweg 2, 2333 CA Leiden, The Netherlands

[3] Research Center for Electronic and Optical Materials, National Institute for Materials Science, 1-1 Namiki, Tsukuba, 305-0044, Japan

[†] Contributed equally

* Corresponding Authors

S1. Theory of vibronic coupling

S2. Methods section

S3. Computational workflow

S4. Additional results of the quantum chemical calculations

S1. Theory of vibronic coupling

We will briefly remind the reader of vibronic coupling and of its importance in chemical analysis and cryogenic single-molecule spectroscopy. As a simplifying hypothesis, we assume that only the minimum of the harmonic potential of each mode changes (slightly) upon an electronic transition from the excited to the ground electronic state, i.e., that the vibration frequency does not change. This approximation, called linear vibronic coupling, works well for most intramolecular vibration modes of aromatic molecules. Figure S1a shows a cut of the potential hypersurfaces of the ground (S_0) and excited (S_1) state along the vibrational coordinate, x, of a single vibration mode. At liquid-helium temperature, fluorescence proceeds from the lowest vibronic state of the excited state S_1 to vibrationally excited levels of the ground state S_0 (see downward arrows in Figure S1a). The corresponding lines in the fluorescence spectrum will be the purely electronic transition (hereafter called 0-0 zero-phonon line and denoted with a yellow arrow, or ZPL for short) and a series of vibronic components at longer wavelengths corresponding to the creation of 1 (dominantly), 2 or more quanta of vibration (arrows of respectively orange and red color).

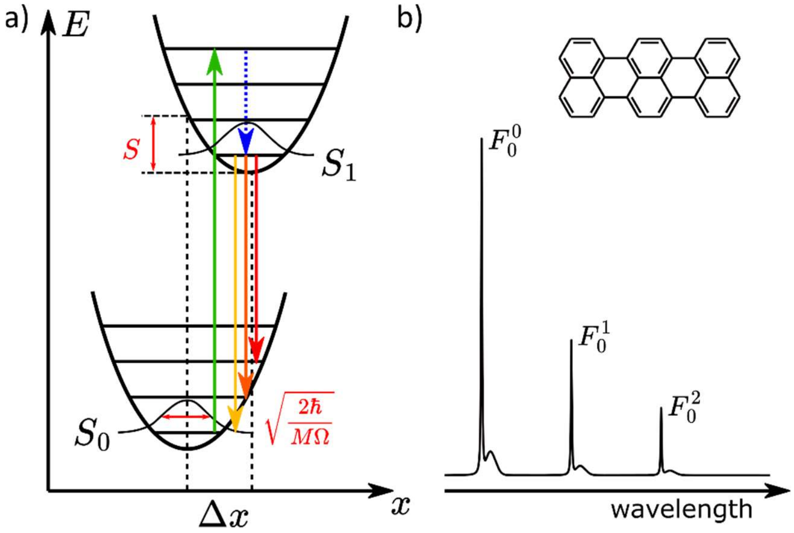

***Figure S1**: a) Schematic harmonic potentials of the ground and excited molecular electronic states, with associated vibrational levels for a single vibration mode (mode coordinate x) under the linear coupling approximation. The figure displays some quantities used in the text of section S1. The intensity of vibronic bands scales as the overlap of nuclear vibrational wavefunctions and for the 0-1 emission (orange arrow) it increases quadratically with the coordinate displacement Δx, as long as it remains small. The shift Δx can be alternatively represented by the associated energy stabilization S upon relaxation of the ground state geometry in the excited state. b) Schematic structure of terrylene together with a schematic representation of typical emission lines (downward arrows in (a)) whose relative intensities are associated with Franck-Condon factors. The vibronic bands are usually observed to be coupled to phonons by the presence of a phonon sideband, appearing on the red side of each narrow line.*

The intensity of the vibronic lines is given by the overlap of nuclear wavefunctions called Franck-Condon factors. For linear coupling to one mode, the Franck-Condon factor F_0^n for the release of n quanta has a simple expression:

$F_0^n = \frac{\xi^{2n}}{n!} e^{-\xi^2}$, $F_0^0 = e^{-\xi^2}$, with

$$\xi = \frac{\Delta x}{\sigma_x} \ ,$$

where Δx is the position shift of the mode upon electronic excitation, and $\sigma_x = \sqrt{\frac{2\hbar}{M\Omega}}$ characterizes the coordinate's quantum spread in the ground state of the mode (with effective mass M and frequency Ω). The position shift of the mode is often characterized by the ratio of the reorganization energy or Stokes loss S (see Figure S1) to the vibrational quantum $\hbar\Omega$:

$$\xi^2 = \frac{\mathrm{S}}{\hbar\Omega} \ .$$

For several independent modes, and assuming that the mode decomposition does not change upon excitation, the Franck-Condon factors are simply the products of factors for each mode i. The strength of the 0-0 transition is then $\alpha_{\mathrm{FC}} = \exp(-\xi^2)$, with

$$\xi^2 = \sum_i \frac{\mathrm{S}_i}{\hbar\Omega_i} \ .$$

It is usual and convenient to distinguish lattice phonons from intramolecular vibrations. For lattice phonons, the above analysis leads to a ratio of the pure electronic transition intensity I_{ZPL} compared to that I_{PW} of the phonon wing $\alpha_{\mathrm{DW}} = I_{\mathrm{ZPL}}/(I_{\mathrm{ZPL}} + I_{\mathrm{PW}})$ called the Debye-Waller factor. The strength of the combined purely electronic transition free from any phonon or intramolecular vibration, the 0-0 ZPL, is thus a combined Franck-Condon-Debye-Waller factor α_{FCDW}:

$$\alpha_{\mathrm{FCDW}} = \alpha_{\mathrm{FC}}\alpha_{\mathrm{DW}}.$$

In the case of many weakly coupled modes, this factor can be calculated for a non-zero temperature T, as[1]:

$$\alpha_{\mathrm{FCDW}}(T) = \exp\left\{-\sum_i \frac{\mathrm{S}_i}{\hbar\Omega_i} \coth\frac{\hbar\Omega_i}{2k_B T}\right\},$$

where the sums run over all modes, intramolecular and lattice phonons. The very fast decay with temperature of this factor explains why the ZPL can only observed at liquid-helium temperatures in molecular materials, whose vibrations frequencies are low compared to many inorganic materials.

S2. Methods section

Experimental details

The hBN was obtained from two manufacturers: the company HQ-Graphene and the NIMS research institute[2]. Flakes of hBN were retrieved from a single crystal and deposited on a Si/SiO_2 substrate (University Wafer) through the exfoliation method using scotch tape. After exfoliation, the substrates were annealed in a tube oven (Thermcraft) at 750 ˚C for 12 hours in a moderate vacuum (rough pumping) and in the presence of oxygen. Subsequently, some terrylene was deposited on the annealed flakes by sublimation in a vacuum sublimation apparatus. These samples were loaded into the cryostat (Janis SVT-200-5) for measurement. After cooldown with liquid helium, the emission spectra were recorded upon excitation with a laser at 532 nm (Sprout-G15W, Lighthouse Photonics) in a confocal setup equipped with a Horiba IHR-320 spectrometer.

Computational details

All periodic density functional theory (DFT) based geometry optimizations and molecular dynamics simulations were performed within the CP2K.8.2 software package.[3] The electron density was expanded in a hybrid Gaussian and plane wave scheme as is implemented in the Quickstep module within CP2K.8.2.[4] The boron valence electrons were described by the DZVP-MOLOPT-SR-GTH basis set, while the DZVP-MOLOPT-GTH basis set was used for all other atoms.[5] The atom-centered basis set was augmented with plane waves up to a kinetic energy cutoff of 500 Ry. The Brillouin zone was integrated over the Γ-point only. The core electrons were modeled with the PBE-optimized, norm-conserving pseudopotentials developed by Goedecker, Teter and Hutter.[6–8] During the geometry optimizations, the electronic structure was consistently converged to a value of 10^{-7} a.u. Geometry optimizations were carried out with the conjugated gradients algorithm until a precision of 10^{-5} Ha $Bohr^{-1}$ and 10^{-4} Bohr was reached in the root mean squared change of the forces and geometry step size, respectively. The electron density was integrated over a multigrid of size 5. The calculations discussed in the main paper employ the screened hybrid-density functional introduced by Heyd, Scuseria and Ernzerhof (referred to as the HSE06 functional),[9–12] in combination with Grimme's third generation of atom pairwise dispersion corrections with Becke-Johnson damping (D3(BJ)) and a 22 Å cutoff.[13–15] The Hartree-Fock exchange was computed in an auxiliary density matrix using the AUX_FIT cFIT3 basis for all elements.[16]

The employed exchange-correlation functional plus dispersion correction setup has been used successfully in literature to study the electronic structure of polycyclic aromatic hydrocarbons adsorbed on hBN.[17] To assess the effect of the functional and dispersion corrections on the terrylene adsorption distance, additional calculations were performed with the GGA-PBE functional, the hybrid-B3LYP functional and the non-local dispersion-corrected rVV10 functional. The terrylene-hBN adsorption distances, calculated with these functionals are provided in Table S1. These calculations were all performed in a periodic unit cell which was optimized at the PBE level. This PBE optimization resulted in a lattice parameter of 2.51 Å, which is very close to the experimentally observed lattice parameter of 2.50 Å. It was found that the HSE06 functional produces a terrylene-hBN distance in line with other calculations, while producing a more accurate band gap for hBN. Therefore, this functional plus dispersion correction was employed for the calculations in the main paper. Subsequently, also the cell-optimization was performed again at this level of theory, yielding the correct lattice parameter of 2.50 Å.

Molecular dynamics (MD) simulations were performed at the same level of theory as the geometry optimizations with the PBE functional. However, the plane-wave cutoff was lowered to 400 Ry to speed up the calculations. The MD simulations employed a 0.5 fs timestep and were performed within the NVT ensemble by means of the Canonical Sampling Through Velocity Rescaling (CSVR) thermostat with a time constant of 20 fs for equilibration and 150 fs for production runs.[18]

***Table S1**: Interplanar terrylene-hBN distance calculated with four different functionals plus dispersion corrections.*

	PBE + D3	B3LYP + D3BJ	HSE06 + D3BJ*	rVV10
Terrylene-hBN distance (Å)	3.34	3.21	3.25	3.31

* Cell optimization was performed also at this level of theory. Other calculations used the cell optimized at the PBE+D3 level.

The vibrationally resolved fluorescence spectra were calculated with the FCclasses program,[20] within the framework of the adiabatic Hessian approximation. The nuclear dependence of the electronic transition dipole is described only in constant terms (Franck-Condon approximation). As input for the FCclasses program, ground and excited state optimized structures and Hessians, as well as transition dipole moments, were calculated with the Gaussian16.02 software.[21] The terrylene-hBN interface was described using a two-layer ONIOM procedure,[22,23] in which the terrylene was modeled at the DFT level, while the hBN electronic structure was described at the PM3 semi-empirical level.[24] Time-dependent DFT (TDDFT) was employed for the excited state calculations on terrylene. The high level ONIOM layer utilized the B3LYP exchange correlation functional, expanded in a 6-31G(d,p) basis set.[25–28] Again, the D3BJ corrections were included to improve the description of dispersion forces. Notably, a very similar setup has been used in literature to interpret the vibronic spectrum of terrylene in naphthalene crystals.[29] Both ground and excited state ONIOM-based structure optimizations were initiated from the ground state optimized structures, extracted from the previously described periodic DFT calculations. During the ONIOM calculations, the PM3 level hBN layer was constrained except for a set of newly introduced hydrogen atoms, which were added manually to saturate the terminal N and B atoms.

S3. Computational workflow

A two-dimensional, single layer of hBN was optimized in a hexagonal periodic unit cell containing 9 x 9 boron nitride units, amounting to a total of 81 boron and 81 nitrogen atoms. During this procedure, both the unit cell size and interatomic forces in the hBN lattice were relaxed, resulting in a hBN lattice constant of 2.50 Å and a B-N bond length of 1.44 Å. These values are within 0.004 Å agreement with X-ray data reported in literature.[30,31] The preoptimized terrylene molecule was subsequently placed in the unit cell, in a parallel alignment with the surface and an intermolecular distance of ~3.5 Å. The subsequent structure optimization involved only relaxation of the interatomic distances, while the cell dimensions were kept constant.

After optimization of terrylene on the perfect hBN surface, defects were introduced into the hBN monolayer. All single-atom defects were introduced by removing or substituting one atom underneath the adsorbed terrylene molecule and subsequently performing a geometry optimization of the whole terrylene-hBN interface. Separately, an optimization of the hBN monolayer without the terrylene molecule was also performed to calculate the adsorption energy.

The periodic full-DFT optimized ground-state structure of the terrylene-hBN interface was taken as input for the non-periodic ONIOM calculations, where the boron and nitrogen atoms at the box edges were terminated with hydrogen atoms. As the previous full-DFT calculations revealed considerable charge transfer from the V_N and O_N to terrylene, the ONIOM calculations with these defects were initiated with the excess electron transferred from the low-level (PM3) hBN layer to the high-level (TD-DFT) terrylene layer. Similarly, calculations with the V_B vacancy were initiated with one electron transferred from the high level layer to the low level layer. We note here that the defect-induced charge transfer results in the ground and excited states of interest being doublet states. In the presence of defects, additional electronic excitations with very low oscillator strength ($f < 0.05$) appear between the relevant ground and excited states. Throughout this study, we consistently identified the excited state of interest by verifying that the excitation predominantly involved the HOMO to LUMO transition in terrylene, and that the oscillator strength remains large ($f \approx 0.8$). On the perfect monolayer, both the S_0 and S_1 states of terrylene molecules were found to adsorb to the hBN with a bond distance of 3.03 Å (see table S3). This is slightly shorter than in the full-DFT calculation (3.25 Å, see table S3), however, we do not expect that this will affect significantly the fluorescence spectrum. It is known that the wavenumbers for peaks further away from the 0-0 ZPL are increasingly overestimated.[32] Therefore, all the spectra have been corrected with a linear scaling function of the form $y = ax + b$, where the a and b parameters were fitted such that the spectrum of terrylene in vacuum reproduced accurately the peaks at 247 and 1817 cm^{-1} observed in the spectrum presented in Figure 1a. Fitting the spectrum for terrylene on pristine hBN yielded parameters of $a = 0.97655035$ and $b = 4.1319276$, which have subsequently also been used to scale all the other spectra.

Interestingly, transitions at 33 and 276 cm^{-1} are observed for terrylene in vacuum but not for terrylene on hBN. These transitions are associated with a slow, out of plane torsional mode that is inhibited in the adsorbed system (Figure S6, mode 2). Some of the experimental single-molecule fluorescence spectra show lines in the same region (see for instance, Figure 1, molecule A, line at 281 cm^{-1}).

S4. Additional results of the quantum chemical calculations

To assess the charge transfer from hBN to terrylene in the presence of different defects, we calculated the partial charge on terrylene by summing the partial charges of all atoms belonging to terrylene. We have performed this analysis with Mulliken charges, Hirshfeld charges and charges obtained from the Intrinsic Atomic Orbital Analysis (IOA).[19] The obtained values are provided in Table S2. All three analysis methods produce overall the same trend in charge transfer from hBN to terrylene. Only an almost rigid shift in the absolute values is observed from one method to another.

Table S2*: Partial charges on terrylene calculated with different charge analysis methods.*

Defect site	Mulliken charge on Terrylene (e^- units)	IOA charges on Terrylene (e^- units)	Hirshfeld charge on Terrylene (e^- units)
Pristine hBN	0.02	0.16	0.30
O_N defect	−0.70	−0.59	−0.36
O_B defect	0.01	0.14	0.32
V_N vacancy	−0.68	−0.57	−0.36
V_B vacancy	0.83	1.06	1.11
V_{BN} vacancy	0.02	0.16	0.29

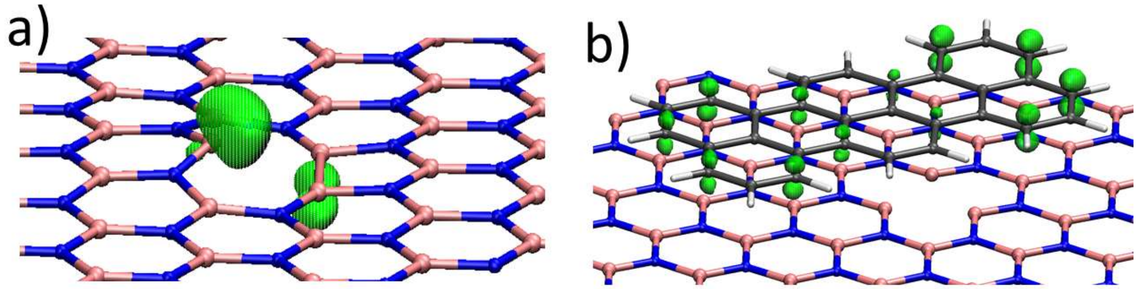

Figure S2. *Spin density of hBN with V_N vacancy (a) and spin density of terrylene chemisorbed at the V_N vacancy (b). Isosurfaces are plotted at 0.01 a.u.*

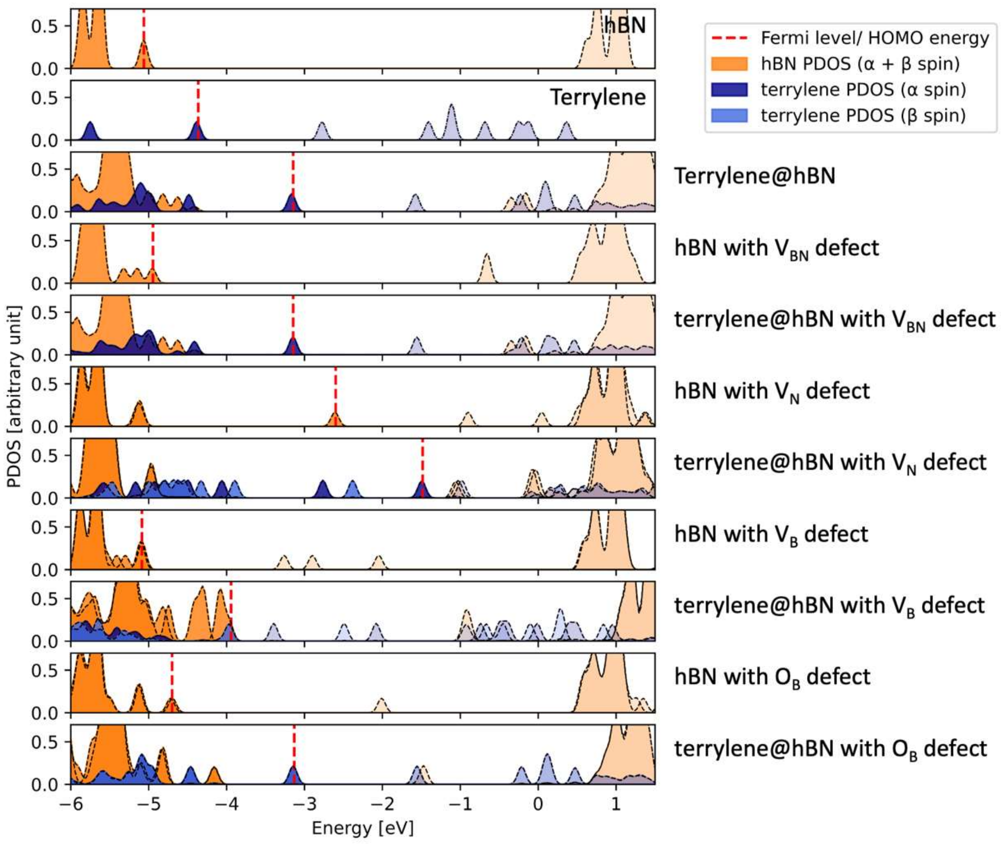

Figure S3. *Projected density of states (PDOS) of pristine hBN and hBN with the V_N, V_B, V_{BN}, O_B defects, with and without chemisorbed terrylene. The plotted density of states were computed in CP2K at the HSE06+D3(BJ)-level and include a Gaussian broadening of 0.05 eV. Bands with transparent colors correspond to unoccupied levels.*

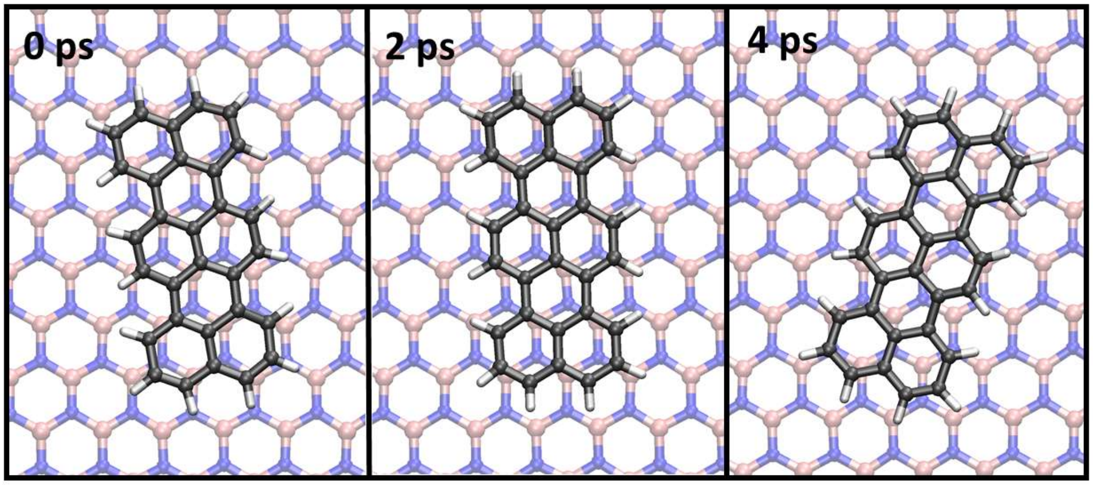

Figure S4. *Snapshots from a DFT-based (PBE+D3(BJ)) molecular dynamics trajectory of terrylene on pristine hBN. The simulation was performed at 300 K. Snapshots are taken at 0, 2 and 4 ps after the start of the production run and show that the terrylene molecule is free to diffuse over the surface.*

Table S3: Terrylene-hBN distances (Å), calculated with full-DFT (HSE06+D3BJ) and with the ONIOM(B3LYP+D3BJ/PM3) approach in the ground and excited states.

	HSE06+D3BJ ground state	ONIOM(B3LYP+D3BJ/PM3) ground state	ONIOM(B3LYP+D3BJ/PM3) excited state
hBN	3.25	3.03	3.03
V_N	3.03	3.00	3.00
V_B	3.20	3.04	3.04
O_N	3.03	3.01	3.01

Table S4: Interpretation of the simulated vibrationally resolved fluorescence spectrum for D_{2h} terrylene in vacuum.

**hv (cm^{-1})**	**hv (cm^{-1})[a]**	**Intensity (a.u.)**	**Assignment**	**Symmetry**
0.0	0.0	1.000	0-0 ZPL	
26.6	30.1	0.026	1^2	Ag (Au^2)
30.1	33.5	0.047	2^2	Ag ($B1g^2$)
248.7	247.0	0.348	11^1	Ag
497.4	489.8	0.060	11^2	Ag
549.2	540.4	0.053	28^1	Ag
593.8	583.0	0.033	33^1	Ag
797.9	783.3	0.018	28^1, 11^1	Ag
842.5	826.9	0.012	33^1, 28^1	Ag
1313.4	1286.7	0.104	86^1	Ag
1348.0	1320.5	0.069	91^1	Ag
1396.4	1367.8	0.023	94^1	Ag
1407.6	1378.7	0.046	96^1	Ag
1562.1	1529.6	0.034	86^1, 11^1	Ag
1596.7	1563.3	0.024	91^1, 11^1	Ag
1608.2	1575.2	0.118	108^1	Ag
1656.3	1621.6	0.015	96^1, 11^1	Ag
1856.4	1818.0	0.039	108^1, 11^1	Ag
2922.2	2857.8	0.011	108^1, 86^1	Ag

[a] scaled with parameters (a, b): [0.97655035 4.1319276]

Table S5: Interpretation of the simulated vibrationally resolved fluorescence spectrum for D_{2h} terrylene on hBN.

hν (cm^{-1})	hν (cm^{-1})[a]	Intensity (a.u.)	Assignment	Symmetry
0.0	0.0	1.000	0-0 ZPL	
249.3	247.6	0.346	17^1	Ag
498.6	491.0	0.059	17^2	Ag
549.9	541.2	0.052	34^1	Ag
594.3	584.5	0.034	39^1	Ag
799.2	784.6	0.018	34^1, 17^1	Ag
843.6	827.9	0.012	39^1, 17^1	Ag
1310.0	1284.4	0.111	92^1	Ag
1345.2	1317.8	0.077	97^1	Ag
1390.4	1361.9	0.021	100^1	Ag
1402.2	1373.5	0.043	101^1	Ag
1559.0	1526.9	0.036	92^1, 17^1	Ag
1594.5	1561.2	0.024	97^1, 17^1	Ag
1603.2	1569.8	0.118	114^1	Ag
1651.5	1616.9	0.014	101^1, 17^1	Ag
1852.5	1813.2	0.037	114^1, 17^1	Ag
2913.3	2849.1	0.012	114^1, 92^1	Ag

[a] scaled with parameters (a, b): [0.97655035 4.1319276]]

Table S6: Interpretation of the simulated vibrationally resolved fluorescence spectrum for D_{2h} terrylene on hBN + O_N defect.

hν (cm^{-1})	hν (cm^{-1})[a]	Intensity (a.u.)	Assignment	Symmetry
0.0	0.0	1.000	0-0 ZPL	
248.3	246.6	0.068	17^1	Ag
552.7	543.9	0.030	34^1	Ag
594.4	584.6	0.006	39^1	Ag
1362.1	1334.3	0.009	97^1	Ag
1387.9	1359.5	0.020	100^1	Ag
1431.3	1401.9	0.012	103^1	Ag
1622.9	1589.0	0.029	113^1	Ag
1731.0	1694.5	0.007	119^1	Ag
3504.03	3426.0	0.010	121^2	Ag ($B1g^2$)

[a] scaled with parameters (a, b): [0.97655035 4.1319276]]

Table S7: Interpretation of the simulated vibrationally resolved fluorescence spectrum for D_{2h} terrylene on hBN + V_N vacancy.

hν (cm^{-1})	hν (cm^{-1})[a]	Intensity (a.u.)	Assignment	Symmetry
0.0	0.0	1.000	0-0 ZPL	
248.4	246.7	0.069	17^1	Ag
552.7	543.9	0.030	34^1	Ag
594.3	584.5	0.008	39^1	Ag
1362.1	1334.3	0.008	97^1	Ag
1388.9	1360.5	0.019	100^1	Ag
1431.4	1402.0	0.013	103^1	Ag
1624.1	1590.2	0.030	113^1	Ag
1731.2	1694.8	0.008	119^1	Ag
2310.1	2260.0	0.005	121^1, 35^1	Ag (B1g*B1g)
2933.6	2869.0	0.005	121^1, 84^1	Ag (B1g*B1g)
3504.4		0.012	121^2	Ag ($B1g^2$)

[a] scaled with parameters (a, b): [0.97655035 4.1319276]]

Table S8: Interpretation of the simulated vibrationally resolved fluorescence spectrum for D_{2h} terrylene on hBN + V_B vacancy.

hν (cm^{-1})	hν (cm^{-1})[a]	Intensity (a.u.)	Assignment	Symmetry
0.0	0.0	1.000	0-0 ZPL	
250.4	248.6	0.050	17^1	Ag
557.2	548.3	0.018	34^1	Ag
601.1	591.2	0.014	39^1	Ag
1380.1	1351.8	0.006	96^1	Ag
1414.3	1385.3	0.024	100^1	Ag
1455.2	1425.2	0.011	103^1	Ag
1643.0	1608.6	0.026	113^1	Ag
1661.6	1626.8	0.019	55^2	Ag ($B3u^2$)
1734.8	1698.2	0.007	119^1	Ag
2258.2	2209.4	0.008	101^1, 55^1	Ag (B3u*B3u)
2274.4	2225.2	0.032	102^1, 55^1	Ag (B3u*B3u)
2553.7	2497.9	0.017	117^1, 55^1	Ag (B3u*B3u)
2871.0	2807.8	0.016	102^1, 101^1	Ag (B3u*B3u)
2887.2	2823.6	0.007	102^1	Ag ($B3u^2$)
3150.3	3080.5	0.006	117^1, 101^1	Ag (B3u*B3u)
3166.4	3096.3	0.017	117^1, 102^1	Ag (B3u*B3u)

[a] scaled with parameters (a, b): [0.97655035 4.1319276]]

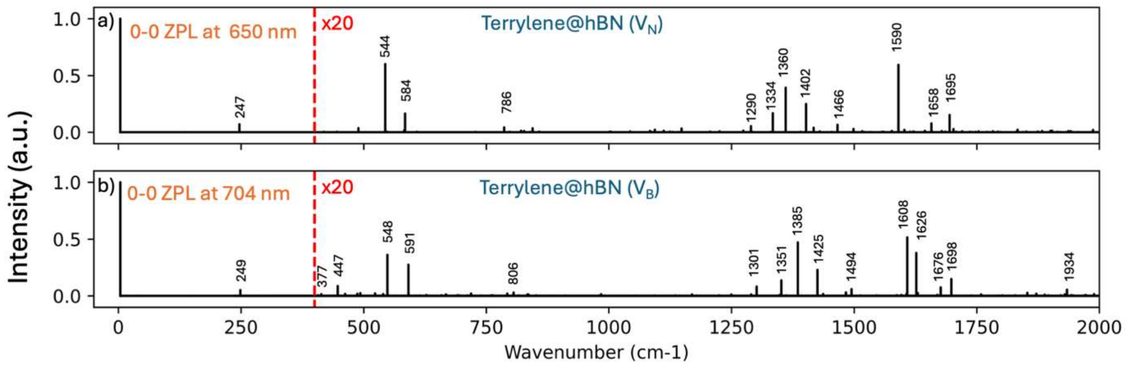

Figure S5. *Computed vibrationally resolved fluorescence spectra of terrylene on the V_N defect (a), and on the V_B defect (b). Both spectra are shifted such that their respective 0-0 ZPL is centered at 0. The spectra are corrected with a linear scaling function with parameters 0.977 and 4.132, as suggested by Palafox.*[32] *All intensities are scaled such that the intensity of the 0-0 ZPL is 1. The intensities of the peaks observed above 400 cm^{-1} are multiplied by a factor 20.*

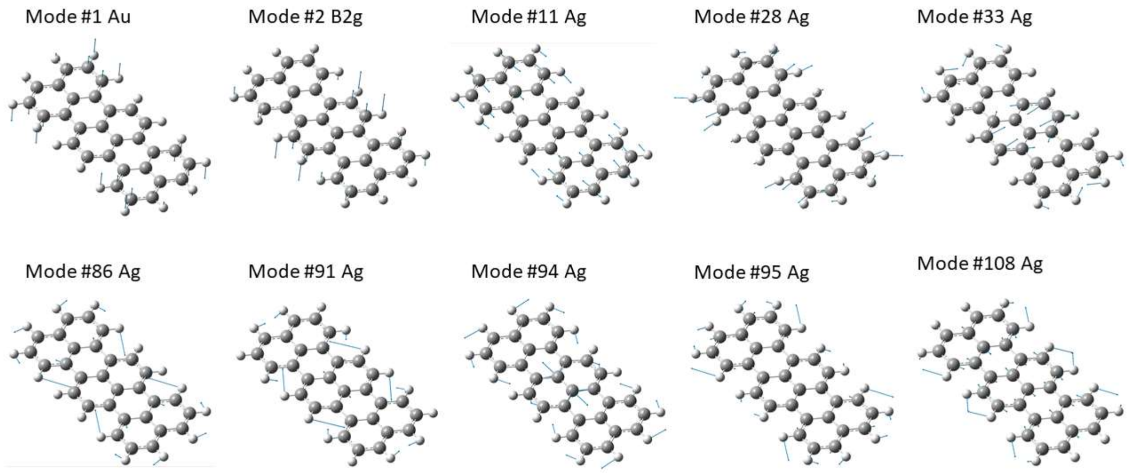

Figure S6. *Visualization of the most prominent vibrational modes observed in the $S_1 \rightarrow S_0$ vibrationally resolved fluorescence spectrum for terrylene in vacuum.*

References

(1) Rebane, K. K. *Impurity Spectra of Solids*; Plenum New York, 1970.

(2) Watanabe, K.; Taniguchi, T.; Kanda, H. Direct-Bandgap Properties and Evidence for Ultraviolet Lasing of Hexagonal Boron Nitride Single Crystal. *Nature Mater* **2004**, *3* (6), 404–409. https://doi.org/10.1038/nmat1134.

(3) Kühne, T. D.; Iannuzzi, M.; Del Ben, M.; Rybkin, V. V.; Seewald, P.; Stein, F.; Laino, T.; Khaliullin, R. Z.; Schütt, O.; Schiffmann, F.; Golze, D.; Wilhelm, J.; Chulkov, S.; Bani-Hashemian, M. H.; Weber, V.; Borštnik, U.; Taillefumier, M.; Jakobovits, A. S.; Lazzaro, A.; Pabst, H.; Müller, T.; Schade, R.; Guidon, M.; Andermatt, S.; Holmberg, N.; Schenter, G. K.; Hehn, A.; Bussy, A.; Belleflamme, F.; Tabacchi, G.; Glöß, A.; Lass, M.; Bethune, I.; Mundy, C. J.; Plessl, C.; Watkins, M.; VandeVondele, J.; Krack, M.; Hutter, J. CP2K: An Electronic Structure and Molecular Dynamics Software Package - Quickstep: Efficient and Accurate Electronic Structure Calculations. *The Journal of Chemical Physics* **2020**, *152* (19), 194103. https://doi.org/10.1063/5.0007045.

(4) VandeVondele, J.; Krack, M.; Mohamed, F.; Parrinello, M.; Chassaing, T.; Hutter, J. Quickstep: Fast and Accurate Density Functional Calculations Using a Mixed Gaussian and Plane Waves Approach. *Computer Physics Communications* **2005**, *167* (2), 103–128. https://doi.org/10.1016/j.cpc.2004.12.014.

(5) VandeVondele, J.; Hutter, J. Gaussian Basis Sets for Accurate Calculations on Molecular Systems in Gas and Condensed Phases. *The Journal of Chemical Physics* **2007**, *127* (11), 114105. https://doi.org/10.1063/1.2770708.

(6) Goedecker, S.; Teter, M.; Hutter, J. Separable Dual-Space Gaussian Pseudopotentials. *Phys. Rev. B* **1996**, *54* (3), 1703–1710. https://doi.org/10.1103/PhysRevB.54.1703.

(7) Hartwigsen, C.; Goedecker, S.; Hutter, J. Relativistic Separable Dual-Space Gaussian Pseudopotentials from H to Rn. *Phys. Rev. B* **1998**, *58* (7), 3641–3662. https://doi.org/10.1103/PhysRevB.58.3641.

(8) Krack, M. Pseudopotentials for H to Kr Optimized for Gradient-Corrected Exchange-Correlation Functionals. *Theor Chem Acc* **2005**, *114* (1–3), 145–152. https://doi.org/10.1007/s00214-005-0655-y.

(9) Heyd, J.; Scuseria, G. E.; Ernzerhof, M. Hybrid Functionals Based on a Screened Coulomb Potential. *The Journal of Chemical Physics* **2003**, *118* (18), 8207–8215. https://doi.org/10.1063/1.1564060.

(10) Heyd, J.; Scuseria, G. E. Efficient Hybrid Density Functional Calculations in Solids: Assessment of the Heyd–Scuseria–Ernzerhof Screened Coulomb Hybrid Functional. *The Journal of Chemical Physics* **2004**, *121* (3), 1187–1192. https://doi.org/10.1063/1.1760074.

(11) Heyd, J.; Peralta, J. E.; Scuseria, G. E.; Martin, R. L. Energy Band Gaps and Lattice Parameters Evaluated with the Heyd-Scuseria-Ernzerhof Screened Hybrid Functional. *The Journal of Chemical Physics* **2005**, *123* (17), 174101. https://doi.org/10.1063/1.2085170.

(12) Heyd, J.; Scuseria, G. E.; Ernzerhof, M. Erratum: "Hybrid Functionals Based on a Screened Coulomb Potential" [J. Chem. Phys. 118, 8207 (2003)]. *The Journal of Chemical Physics* **2006**, *124* (21), 219906. https://doi.org/10.1063/1.2204597.

(13) Grimme, S. Accurate Description of van Der Waals Complexes by Density Functional Theory Including Empirical Corrections. *J. Comput. Chem.* **2004**, *25* (12), 1463–1473. https://doi.org/10.1002/jcc.20078.

(14) Grimme, S. Density Functional Theory with London Dispersion Corrections. *Wiley Interdisciplinary Reviews: Computational Molecular Science* **2011**, *1* (2), 211–228. https://doi.org/10.1002/wcms.30.

(15) Grimme, S.; Ehrlich, S.; Goerigk, L. Effect of the Damping Function in Dispersion Corrected Density Functional Theory. *Journal of Computational Chemistry* **2011**, *32* (7), 1456–1465. https://doi.org/10.1002/jcc.21759.

(16) Guidon, M.; Hutter, J.; VandeVondele, J. Auxiliary Density Matrix Methods for Hartree−Fock Exchange Calculations. *J. Chem. Theory Comput.* **2010**, *6* (8), 2348–2364. https://doi.org/10.1021/ct1002225.

(17) Melani, G.; Guerrero-Felipe, J. P.; Valencia, A. M.; Krumland, J.; Cocchi, C.; Iannuzzi, M. Donors, Acceptors, and a Bit of Aromatics: Electronic Interactions of Molecular Adsorbates on hBN and MoS $_2$ Monolayers. *Phys. Chem. Chem. Phys.* **2022**, *24* (27), 16671–16679. https://doi.org/10.1039/D2CP01502A.

(18) Bussi, G.; Donadio, D.; Parrinello, M. Canonical Sampling through Velocity Rescaling. *The Journal of Chemical Physics* **2007**, *126* (1), 014101. https://doi.org/10.1063/1.2408420.

(19) Knizia, G. Intrinsic Atomic Orbitals: An Unbiased Bridge between Quantum Theory and Chemical Concepts. *J. Chem. Theory Comput.* **2013**, *9* (11), 4834–4843. https://doi.org/10.1021/ct400687b.

(20) Cerezo, J.; Santoro, F. *FCclasses3* : Vibrationally-resolved Spectra Simulated at the Edge of the Harmonic Approximation. *J Comput Chem* **2023**, *44* (4), 626–643. https://doi.org/10.1002/jcc.27027.

(21) 10. Frisch, M. J.; Trucks, G. W.; Schlegel, H. B.; Scuseria, G. E.; Robb, M. A.; Cheeseman, J. R.; Scalmani, G.; Barone, V.; Petersson, G. A.; Nakatsuji, H.; Li, X.; Caricato, M.; Marenich, A. V.; Bloino, J.; Janesko, B. G.; Gomperts, R.; Mennucci, B.; Hr, D. J. ; Gaussian 16, 2016.

(22) Dapprich, S.; Komaromi, I.; Byun, K. S.; Morokuma, K.; Frisch, M. J. A New ONIOM Implementation in Gaussian98. Part I. The Calculation of Energies, Gradients, Vibrational Frequencies and Electric FIeld Derivatives ☆. **1999**.

(23) Chung, L. W.; Sameera, W. M. C.; Ramozzi, R.; Page, A. J.; Hatanaka, M.; Petrova, G. P.; Harris, T. V.; Li, X.; Ke, Z.; Liu, F.; Li, H.-B.; Ding, L.; Morokuma, K. The ONIOM Method and Its Applications. *Chem. Rev.* **2015**, *115* (12), 5678–5796. https://doi.org/10.1021/cr5004419.

(24) Stewart, J. J. P. Optimization of Parameters for Semiempirical Methods V: Modification of NDDO Approximations and Application to 70 Elements. *J Mol Model* **2007**, *13* (12), 1173–1213. https://doi.org/10.1007/s00894-007-0233-4.

(25) Becke, A. D. Density-Functional Exchange-Energy Approximation with Correct Asymptotic Behavior. *Phys. Rev. A.* **1988**, *38*, 3098.

(26) Becke, A. D. A New Mixing of Hartree-Fock and Local Density-Functional Theories. *J. Chem. Phys.* **1993**, *98* (2), 1372–1377. https://doi.org/10.1063/1.464304.

(27) Vosko, S. H.; Wilk, L.; Nusair, M. Accurate Spin-Dependent Electron Liquid Correlation Energies for Local Spin Density Calculations: A Critical Analysis. *Can. J. Phys.* **1980**, *58* (8), 1200–1211. https://doi.org/10.1139/p80-159.

(28) Stephen, P. J.; Devlin, F. J.; Chabalowski, C. F.; Frisch, M. J. Ab Initio Calculation of Vibrational Absorption. *J. Phys. Chem.* **1994**, *98* (45), 11623–11627.

(29) Deperasińska, I.; Kozankiewicz, B. Non-Planar Distortion of Terrylene Molecules in a Naphthalene Crystal. *Chemical Physics Letters* **2017**, *684*, 208–211. https://doi.org/10.1016/j.cplett.2017.06.043.

(30) Lynch, R. W.; Drickamer, H. G. Effect of High Pressure on the Lattice Parameters of Diamond, Graphite, and Hexagonal Boron Nitride. *The Journal of Chemical Physics* **1966**, *44* (1), 181–184. https://doi.org/10.1063/1.1726442.

(31) Sichel, E. K.; Miller, R. E.; Abrahams, M. S.; Buiocchi, C. J. Heat Capacity and Thermal Conductivity of Hexagonal Pyrolytic Boron Nitride. *Phys. Rev. B* **1976**, *13* (10), 4607–4611. https://doi.org/10.1103/PhysRevB.13.4607.

(32) Palafox, M. A. DFT Computations on Vibrational Spectra: Scaling Procedures to Improve the Wavenumbers. *Physical Sciences Reviews* **2018**, *3* (6). https://doi.org/10.1515/psr-2017-0184.
