# Supplementary material for: Charge Transfer-Induced Weakening of Vibronic Coupling for Single Terrylene Molecules Adsorbed onto Hexagonal Boron Nitride
Source: J Phys Chem Lett. 2024 Dec 30;16(1):349–56. doi: 10.1021/acs.jpclett.4c02899 (PMC11726798; doi:10.1021/acs.jpclett.4c02899)
Supplement: Supplementary file 2 — jz4c02899_si_002.pdf [file jz4c02899_si_002.pdf]

jz-2024-02899t.R1

Name: Peer Review Information for "Charge Transfer-Induced Weakening of Vibronic Coupling for Single Terrylene Molecules Adsorbed onto hBN"

First Round of Reviewer Comments

Reviewer: 1

Comments to the Author
Hexagonal boron-nitride (hBN) can serve as a host matrix that may improve the photostability of fluorescent molecules. This has been shown with fluorescent terrylene molecules deposited on hBN exhibiting remarkable, increased photostability at room temperature by Han et al. in 2021 (Ref. 36). In conjunction to the author group's previous work (Ref. 4), the current work extended the earlier studies and uncovered terrylene molecules on the surface of hBN with red-shifted 0-0 ZPL transitions showing weaker vibronic coupling. The experimental findings were accompanied by DFT calculations of the physisorption of terrylene to pristine hBN and selected defect sites (boron or nitrogen vacancies or oxygen substitutions). They found through DFT calculations that terrylene has higher binding affinity to certain defect sites compared to pristine hBN. Based on the DFT Mulliken charges on terrylene, the authors further ascribe experimental observations to terrylene molecules physisorbed on defect sites with electron or hole transfer characters. In this reviewer's view, ascribing the observed single-molecules to those interacting with defects makes sense, in particular in view of the grouping of the observed 0-0 ZPL (Fig. 1e) which strongly suggest a physical original of discrete nature. Overall, this is an interesting fundamental work and adds important new insights to the adsorbate/hBN system.

There are just few questions which will help with the presentation if they can be clarified:

1. It is known that Mulliken charges alone could misrepresent the actual charges (at least on the level of theory) on the designated atom or molecule because they strongly depend on the basis set or functional used. It would be helpful to also list natural orbital charge analysis. Listing both should help with the interpretation and the readers will have a balanced view of the results.
2. It is also curious if Stark effect can be ignored under the experimental conditions. If so, perhaps the authors would consider stating it clearly in the text with a small discussion.
3. If the Stark effect cannot be ignored, which will be consistent with the idea that the observed terrylene molecules tend to interact with defect sites with charge-transfer

characteristics. It is also consistent with the observed emission grouping in Fig. 1e. (3a) Could vibration Stark effect help to explain the trend seen in Fig. 1g? (3b) Given that charge transfer could be involved, should the quantum chemical calculations include such scenarios as to provide estimates for vibrational Stark effect?

Reviewer: 2

Comments to the Author
The manuscript studies the vibrational spectra of terrylene molecules on hBN by combining single molecule fluorescence measurements with DFT based calculations.
The key result is the strong variation in the intensity of the measured vibrational spectra which are of interest for developing efficient emission of single photons. Measurements are rationalised by the calculations at different defect sites, in terms of the defect absorption energy, the coincidence of the defect level and the LUMO of terrylene and charge transfer.
Overall, the manuscript is clearly and concisely written. The methodology is well justified and the SI provides all needed details (at least for the calculation part, I am expert on). The material is well divided between the main manuscript (key results) and the SI (details and additional results).
The experimental results, the quantum chemical explanation, and the reduced vibronic coupling mechanism are relevant for the broader physical chemistry community and I thus believe the manuscript merit publication in the Journal of Physical Chemistry Letter.

Author's Response to Peer Review Comments:

Response Letter for Manuscript ID jz-2024-02899t

[1] For clarity, we report in full the reviewer comments in *italic* font.
[2] Our response is marked as "Author reply".

[3] We also reproduce the changes made in the manuscript specifying the page number and mark them as "Changes"

Reviewer(s)' Comments to Author:

Reviewer: 1

Recommendation: This paper is publishable subject to minor revisions noted. Further review is not needed.

Comments:

Hexagonal boron-nitride (hBN) can serve as a host matrix that may improve the photostability of fluorescent molecules. This has been shown with fluorescent terrylene molecules deposited on hBN exhibiting remarkable, increased photostability at room temperature by Han et al. in 2021 (Ref. 36). In conjunction to the author group's previous work (Ref. 4), the current work extended the earlier studies and uncovered terrylene molecules on the surface of hBN with red-shifted 0-0 ZPL transitions showing weaker vibronic coupling. The experimental findings were accompanied by DFT calculations of the physisorption of terrylene to pristine hBN and selected defect sites (boron or nitrogen vacancies or oxygen substitutions). They found through DFT calculations that terrylene has higher binding affinity to certain defect sites compared to pristine hBN. Based on the DFT Mulliken charges on terrylene, the authors further ascribe experimental observations to terrylene molecules physisorbed on defect sites with electron or hole transfer characters. In this reviewer's view, ascribing the observed single-molecules to those interacting with defects makes sense, in particular in view of the grouping of the observed 0-0 ZPL (Fig. 1e) which strongly suggest a physical original of discrete nature. Overall, this is an interesting fundamental work and adds important new insights to the adsorbate/hBN system.

Author reply

We thank the Reviewer for the positive assessment of our work and for the constructive comments and suggestions that we have taken into account in the revised version of our manuscript.

There are just few questions which will help with the presentation if they can be clarified:

1. *It is known that Mulliken charges alone could misrepresent the actual charges (at least on the level of theory) on the designated atom or molecule because they strongly depend on the basis set or functional used. It would be helpful to also list natural orbital charge analysis. Listing both should help with the interpretation and the readers will have a balanced view of the results.*

Author reply:

We agree that the Mulliken charges are often sensitive to the computational setup. However, in this work we do not look at specific atomic charges, but rather at the sum over all atomic contributions to the total charge of the terrylene and hBN fragments. We expect that the overall charge on the molecular fragments is captured more accurately. Nevertheless, we agree with the reviewer that reporting the charges calculated with different charge analysis methods would benefit the manuscript.

We have therefore included in the SI a table (Table S2) that reports, for all defects, the Mulliken charges, Intrinsic Atomic Orbital (IAO) charges and Hirshfeld charges. All three analysis methods produce overall the same trend in charge transfer from hBN to terrylene. Only an almost rigid shift in the absolute values is observed from one method to another. We can thus conclude that the main message of the manuscript is not affected by the specific choice of charge analysis method.

Changes: SI, page S7

To assess the charge transfer from hBN to terrylene in the presence of different defects, we calculated the partial charge on terrylene by summing the partial charges of all atoms belonging to terrylene. We have performed this analysis with Mulliken charges, Hirshfeld charges and charges obtained from the Intrinsic Atomic Orbital Analysis (IOA).[19] The obtained values are provided in Table S2. All three analysis methods produce overall

the same trend in charge transfer from hBN to terrylene. Only an almost rigid shift in the absolute values is observed from one method to another.

Table S2: *Partial charges on terrylene calculated with different charge analysis methods.*

Defect site	Mulliken charge on Terrylene (e^- units)	IOA charges on Ter-rylene (e^- units)	Hirshfeld charge on Terrylene (e^- units)
Pristine hBN	0.02	0.16	0.30
O_N defect	−0.70	−0.59	−0.36
O_B defect	0.01	0.14	0.32
V_N vacancy	−0.68	−0.57	−0.36
V_B vacancy	0.83	1.06	1.11
V_{BN} vacancy	0.02	0.16	0.29

Changes: page 6 of the manuscript

The charge analysis has also been performed using Hirshfeld and Intrinsic Orbital Analysis charges, which show the same trend and are provided in the SI, Table S2.

2. *It is also curious if Stark effect can be ignored under the experimental conditions. If so, perhaps the authors would consider stating it clearly in the text with a small discussion.*

Author Reply:

There are no external electric fields applied in the experiment that could account for the observed 0-0 ZPL shift. The reviewer might, however, be referring to an internal Stark shift, which was observed by deprotonation of phthalocyanine (Vasilev et al. Nature Comm **2022**, 13 (1), 677). In their work, the additional charge(s) remained confined on the molecule itself. In our calculations, the “Stark effect” by extra charge in the hybrid system of terrylene with the defect in the substrate is implicitly included in the DFT simulation.

We have added a paragraph in the manuscript referring to the paper of Vasilev et al. (Ref. 41) and comparing our system to theirs.

Changes: page 10 of the manuscript

In the work of Vasilev et al.,[41] large shifts of a 0-0 ZPL were also observed upon localized charging of a phthalocyanine molecule through deprotonation. These shifts were interpreted as an 'internal Stark effect'. In our case, the interaction of terrylene with a particular hBN defect also leads to charge transfer from the defect to the aromatic molecule, and therefore to local electric fields shifting the electronic transition. These effects are naturally included in the DFT calculations that reproduce the redshift of the 0-0 ZPL.

3. *If the Stark effect cannot be ignored, which will be consistent with the idea that the observed terrylene molecules tend to interact with defect sites with charge-transfer characteristics. It is also consistent with the observed emission grouping in Fig. 1e. (3a) Could vibration Stark effect help to explain the trend seen in Fig. 1g? (3b) Given that charge transfer could be involved, should the quantum chemical calculations include such scenarios as to provide estimates for vibrational Stark effect?*

Author reply:

In line with our response for comment 2, we also note that the additional charge may affect the vibrational energies in the hybrid system of terrylene and the substrate defect. This effect is also reproduced by the calculation. We have added a few comments in the manuscript to further clarify how the vibrational energies may be affected by charge transfer.

Changes: page 10 of the manuscript

In a similar manner, the change in vibronic coupling intensity, which we observe experimentally and confirm through DFT calculations, could be interpreted as a vibrational Stark effect, i.e., a change of coupling due to charge transfer-induced local fields.

Changes: page 8 of the manuscript

Similarly, one can expect that populating the LUMO orbital may affect the spectral position of the long-axis vibration observed around ≈250 cm-1.

Changes: page 8 of the manuscript

We note that the terrylene HOMO-LUMO gap decreases upon charge transfer. This aligns with the redshift of the 0-0 ZPL that was observed for specific terrylene molecules in the single molecule fluorescence experiments.

Additional Questions:

Urgency: High

Significance: High

Novelty: High

Scholarly Presentation: Top 10%

Is the paper likely to interest a substantial number of physical chemists, not just specialists working in the authors' area of research?: Yes

Reviewer: 2

Recommendation: This paper represents a significant new contribution and should be published as is.

Comments:

The manuscript studies the vibrational spectra of terrylene molecules on hBN by combining single molecule fluorescence measurements with DFT based calculations.

The key result is the strong variation in the intensity of the measured vibrational spectra which are of interest for developing efficient emission of single photons. Measurements are rationalised by the calculations at different defect sites, in terms of the defect absorption energy, the coincidence of the defect level and the LUMO of terrylene and charge transfer.

Overall, the manuscript is clearly and concisely written. The methodology is well justified and the SI provides all needed details (at least for the calculation part, I am expert on). The material is well divided between the main manuscript (key results) and the SI (details and additional results).

The experimental results, the quantum chemical explanation, and the reduced vibronic coupling mechanism are relevant for the broader physical chemistry community and I thus believe the manuscript merit publication in the Journal of Physical Chemistry Letter.

Additional Questions:

Urgency: High

Significance: High

Novelty: High

Scholarly Presentation: High

Is the paper likely to interest a substantial number of physical chemists, not just specialists working in the authors' area of research?: Yes

Author reply

We thank the Reviewer for the positive comments on our work.
